# Simple Estimation of the Endolymphatic Volume Ratio after Intravenous Administration of a Single-dose of Gadolinium Contrast

**DOI:** 10.2463/mrms.mp.2015-0175

**Published:** 2016-03-21

**Authors:** Shinji NAGANAWA, Mai KANOU, Toshio OHASHI, Kayao KUNO, Michihiko SONE

**Affiliations:** 1 Department of Radiology, Nagoya University Graduate School of Medicine, 65 Tsurumai-cho, Shouwa-ku, Nagoya 466-8550, Japan; 2 Department of Radiology, Kamiiida Daiichi General Hospital; 3 Department of Otorhinolaryngology, Kamiiida Daiichi General Hospital; 4 Department of Otorhinolaryngology, Nagoya University Graduate School of Medicine

**Keywords:** magnetic resonance imaging, endolymphatic hydrops, temporal bone disease, volume quantification

## Abstract

**Purpose:**

To evaluate the feasibility of a simple estimation for the endolymphatic volume ratio (endolymph volume/total lymph volume = %EL_volume_) from an area ratio obtained from only one slice (%EL_1slice_) or from three slices (%EL_3slices_). The %EL_volume,_ calculated from a time-consuming measurement on all magnetic resonance (MR) slices, was compared to the %EL_1slice_ and the %EL_3slices_.

**Methods:**

In 40 ears of 20 patients with a clinical suspicion of endolymphatic hydrops, MR imaging was performed 4 hours after intravenous administration of a single dose of gadolinium-based contrast material (IV-SD-GBCM). Using previously reported HYDROPS2-Mi2 MR imaging, the %EL_volume_ values in the cochlea and the vestibule were measured separately by two observers. The correlations between the %EL_1slice_ or the %EL_3slices_ and the %EL_volume_ values were evaluated.

**Results:**

A strong linear correlation was observed between the %EL_volume_ and the %EL_3slices_ or the %EL_1slice_ in the cochlea. The Pearson correlation coefficient (*r*) was 0.968 (3 slices) and 0.965 (1 slice) for observer A, and 0.968 (3 slices) and 0.964 (1 slice) for observer B (*P* < 0.001, for all). A strong linear correlation was also observed between the %EL_volume_ and the %EL_3slices_ or the %EL_1slice_ in the vestibule. The Pearson correlation coefficient (*r*) was 0.980 (3 slices) and 0.953 (1 slice) for observer A, and 0.979 (3 slices) and 0.952 (1 slice) for observer B (*P* < 0.001, for all). The high intra-class correlation coefficients (0.991–0.997) between the endolymph volume ratios by two observers were observed in both the cochlea and the vestibule for values of the %EL_volume_, the %EL_3slices_ and the %EL_1slice_.

**Conclusion:**

The %EL_volume_ might be easily estimated from the %EL_3slices_ or the %EL_1slice_.

## Introduction

Clinical evaluation of endolymphatic hydrops (EHs) using magnetic resonance (MR) imaging has been performed in patients with suspected Menière’s disease at 24 hours after intratympanic administration of gadolinium-based contrast material (IT-GBCM)^1–3^ or at 4 hours after intravenous administration of a single dose of gadolinium-based contrast material (IV-SD-GBCM).^4–7^ Although IT-GBCM usually provides a higher contrast concentration in labyrinth than IV-SD-GBCM, MR imaging by IT-GBCM requires off-label use of the contrast material and the invasive puncture of the tympanic membrane.^3^ Therefore, IV-SD-GBCM is becoming more popular in clinical practice.

To compensate for the lower contrast concentration when using IV-SD-GBCM, multiplication of the T_2_-weighted MR cisternography on HYDROPS (HYbriD of Reversed image Of Positive endolymph signal and native image of positive perilymph Signal) or HYDROPS2 (HYbriD of Reversed image Of MR cisternography and positive Perilymph Signal by heavily T_2_-weighted 3D-FLAIR) images has been developed to increase the contrast-to-noise ratio (CNR) more than 200-fold.^8–11^ A less observer-dependent area quantification method was proposed for imaging of EH after IV-SD-GBCM using multiplied images such as HYDROPS-Mi2 (HYDROPS-Multiplied with heavily T_2_-weighted MR cisternography) and HYDROPS2-Mi2 (HYDROPS2-Multiplied with heavily T_2_-weighted MR cisternography).^10,11^ The high CNR by HYDROPS-Mi2 and HYDROPS2-Mi2 enabled a dramatic reduction in scan time while maintaining an area measurement similar to that obtained by the conventional protocol with a longer scan time previously in use with IV-SD-GBCM.^10–12^

Quantification of the endolymphatic volume ratio is used to deepen the understanding of the pathophysiology for Menière’s disease and as an imaging biomarker to monitor treatment response.^13^ MR imaging after intratympanic contrast application and image processing, including sophisticated machine learning and automated local thresholding, enabled the volumetric quantification of EHs, although this method is rather time-consuming for clinical practice.^13^ Recently, the volume ratio of the endolymph against the total lymph on the images obtained by IV-SD-GBCM was measured by drawing a region of interest (ROI) on all slices including the cochlea or the vestibule in an extension of a previously proposed, less observer-dependent, method of area quantification.^14^ However, to draw ROIs for the cochlea and the vestibule bilaterally on all slices (typically 7–9 slices) is time-consuming. The purpose of this study was to evaluate the feasibility of estimating the endolymphatic volume ratio obtained by measurement on only 1 or 3 slices compared to that obtained by measurement of all slices.

## Materials and Methods

### Patients

Forty ears in 20 patients with a clinical suspicion of EHs were included. Patients underwent an MR examination in the time period from April 2013 to January 2014. MR studies were performed for the evaluation of EHs. Imaging data from these patients had been utilized in the previous study for the total volume quantification.^14^ Experienced otorhinolaryngologists determined the indication for the MR examination. A differential diagnosis of Menière’s disease was based on the guidelines of the American Academy of Ophthalmology and Otolaryngology–Head and Neck Surgery (AAO-HNS).^15^ The patients included 5 men and 15 women with an age range of 41–80 years (median 64). A medical ethics committee approved this retrospective study with a waiver for informed consent.

### MR imaging

All MR imagings were performed using a 3-tesla unit (Skyra, Siemens, Erlangen, Germany) with a 32-channel array head coil. MR scanning was performed 4 hours after a single dose with IV administration (0.2 ml/kg body weight or 0.1 mmol/kg body weight) of gadoteridol (Gd-HP-DO3A: ProHance, Eisai, Tokyo). All patients had an estimated glomerular filtration rate (eGFR) value exceeding 60 mL/min/1.73 m.^2^

According to the clinical protocol used by the hospital for the evaluation of EHs,^10–12^ the patients underwent heavily T_2_-weighted MR cisternography (MRC) for anatomical reference of total lymph fluid and a heavily T_2_-weighted 3D-FLAIR with a 2250-msec inversion time (positive perilymph image, PPI) 4 hours after receiving the IV-SD-GBCM. Parameters were set as previously reported.^10–12,14^ A PPI (15 minutes) and an MRC (3 minutes) were obtained. Table 1 details the scan parameters.

### Image processing

Image processing methods were identical to that used in a previous study.^14^ Briefly, HYDROPS2-Mi2 images were generated as follows: (PPI − 0.04 × MRC) × MRC.

Multiplication by the MRC was used to boost the CNR between the endo- and perilymph while suppressing and stabilizing the background signal from the bone and air.^8^ We employed a constant value of 0.04 according to a recent study^12^ and generated the HYDROPS2 images on the scanner console. For the subtraction, negative signal values were allowed. During this step, no image registration program was applied.

Then, we transferred the MR images to a Mac Book Pro personal computer (Apple Computer Inc., Cupertino, CA, USA) with a free DICOM viewer (OsiriX image software, ver. 5.0.2 32 bit; downloadable at http://www.osirix-viewer.com/index.html), which allowed easy pixel-by-pixel multiplication between the image series.

### Volume and area measurement

**Fig. 1.**
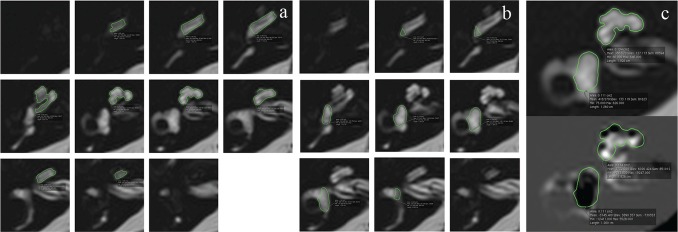
An example of the MRC images with the ROI placement for the cochlea (**a**) and the vestibule (**b**). The number of slices contoured was 7–9 for the cochlea and 5–7 for the vestibule. These ROIs on the MRC images were copied onto the HYDROPS2-Mi2 images (**c**). MRC, magnetic resonance cisternography; ROI, region of interest.

Voxels with a negative value on the HYDROPS2-Mi2 image were estimated as endolymph. In the 40 ears, we measured the percentage of the volume of endolymphatic space in the total lymphatic space (%EL_volume_) for the cochlea and the vestibule quantitatively on the HYDROPS2-Mi2 image according to a previously reported threshold-based method.^10,12^ Two radiological technologists (with experience of 10 and 14 years in MR imaging) contoured the ROI around the cochlea and the vestibule on the MRC slices according to the instructions indicated below and example images (Fig. 1) used in the previous study.^14^ The number of slices that were contoured was between 7–9 for the cochlea and 5–7 for the vestibule. When observers contour the labyrinth, threshold of the full width at half maximum on MRC slices was subjectively employed in practice.

A neuroradiologist with 27 years of experience in MR imaging set up the instructions and example images for contouring. The instructions shown below are a modification of that used for the area measurement.^12^

1. Before starting to contour the cochlea or the vestibule on the MRC, observers should set the image window level and width to 400/1000.
2. For the cochlear ROI, observers should exclude the part where the signal intensity is lower than half of the fully fluid containing voxels due to a partial volume averaging effect on the MRC. Observers should also exclude the cochlear modiolus when drawing the ROI. Observers do not have to exclude the thin osseous spiral lamina. In the connecting part of the basal turn with the vestibule, observers should draw the border at the posterior edge of the osseous spiral lamina.
3. For the vestibular ROI, observers should exclude the semicircular canals and the ampullas when drawing an ROI for the vestibule on the MRC. In the connecting part of the basal turn of the cochlea with the vestibule, observers should draw the border at the posterior edge of the osseous spiral lamina.

The two observers consulted example images when drawing the ROIs. The ROIs drawn on the MRC were copied and pasted onto the HYDROPS2-Mi2 images (Fig. 1). We used the histogram function of OsiriX to count the number of all voxels within the ROI and the number of voxels with a negative signal intensity value (i.e., endolymph) within the ROI.

The ratio of the volume (%) of the endolymphatic space in the entire lymphatic space (%EL_volume_) was defined as: %EL_volume_ = (sum of the number of negative voxels for the endolymph in the ROI of all slices divided by the total number of voxels in the ROIs of all slices) × 100. This method for volume quantification was identical to the previously reported method.^14^

After 10 months, the same two observers performed the area ratio measurements for 1 slice and 3 slices. The cochlea and the vestibule were contoured separately to place an ROI on the MRC. Both observers received the following instructions that were modified from the instructions for the volume measurement^14^:

1. Before starting the contouring of the cochlea or the vestibule on the MRC, set the image window level and width to 400/1000.
2. For the cochlear ROI, select the center slice on which the cochlear modiolus is largest visually. If the size of the modiolus is comparable on 2 or more slices, choose the slice with the largest height of the modiolus. When contouring the cochlea on the MRC, exclude the modiolus when drawing the ROI.
3. For the vestibular ROI, select the lowest slice where the lateral semicircular canal ring is visualized more than 240°, and exclude the semicircular canal and the ampulla when drawing the ROI for the vestibule on the MRC.

The ROI of the cochlear slice was defined to select the middle part of the cochlea, and the ROI of the vestibular slice, to select the middle of the vestibule. The ROIs, drawn on the MRC, were copied and pasted onto the HYDROPS2-Mi2 image. We then used the histogram function of OsiriX to measure the total number of pixels in the ROI and the number of pixels with a negative signal intensity value (i.e., endolymph) in the ROI.

The ratio of the area (%) of the endolymphatic space in the entire lymphatic space (%EL_1slice_) was defined as: %EL_1slice_ = (the number of negative pixels for the endolymph in the ROI divided by the total number of pixels in the ROI) × 100. Then ROIs were also placed on the MRC images one slice up (1 mm up) and one slice down (1 mm down) from the selected center slice of the cochlea and that of the vestibule. The %EL for 3 slices (%EL_3slices_) was defined as %EL_3slices_ = (the sum of the number of negative pixels for the endolymph in the ROIs on the 3 slices divided by the total number of pixels in the ROIs in the 3 slices) × 100.

### Statistical analysis

The correlation between the %EL_volume_ and the %EL_3slices_ or the %EL_1slice_ was evaluated by Pearson correlation coefficient for both the cochlea and the vestibule over all 40 ears. A linear regression line was calculated by simple regression analysis. The intra-class correlation coefficient for the %EL_1slice_, %EL_3slices_, and the %EL_volume_ between the two observers was also evaluated for the cochlea and the vestibule independently.

## Results

**Fig. 2.**
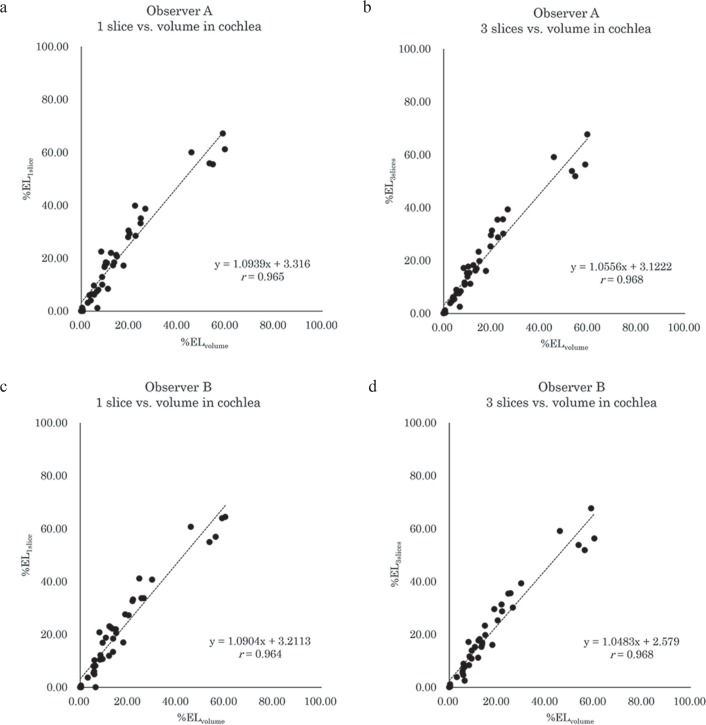
A scattergram of the %EL_volume_ and the %EL_1slice_ or the %EL_3slices_ for the cochlea by observers A and B. A strong linear correlation was observed between the %EL_volume_ and the %EL_1slice_ or the %EL_3slices_ of the cochlea for both observers A (**a**, **b**) and B (**c**, **d**).

A strong linear correlation was observed between the %EL_volume_ and the %EL_1slice_ or the %EL_3slices_ of the cochlea (Fig. 2). The Pearson correlation coefficient (*r*) was 0.968 (3 slices) and 0.965 (1 slice) for observer A, and 0.968 (3 slices) and 0.964 (1 slice) for observer B (*P* < 0.001, for all).

**Fig. 3.**
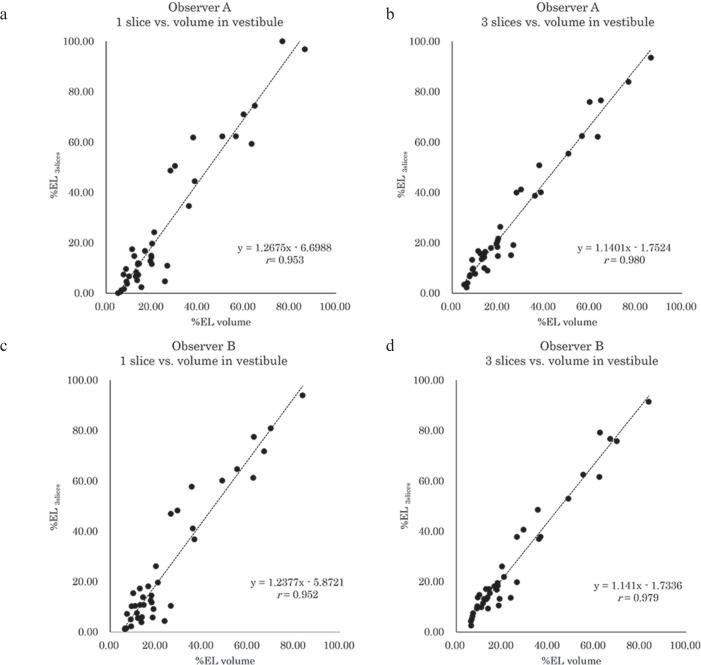
A scattergram of the %EL_volume_ and the %EL_1slice_ or the %EL_3slices_ for the vestibule by observers A and B. A strong linear correlation was observed between the %EL_volume_ and the %EL_1slice_ or the %EL_3slices_ of the vestibule for both observers A (**a**, **b**) and B (**c**, **d**).

A strong linear correlation was also observed between the %EL_volume_ and the %EL_1slice_ or the %EL_3slices_ of the vestibule (Fig. 3). The Pearson correlation coefficient (*r*) was 0.980 (3 slices) and 0.953 (1 slice) for observer A, and 0.979 (3 slices) and 0.952 (1 slice) for observer B (*P* < 0.001, for all).

The intra-class correlation coefficient between the volume ratios by two observers was 0.994 for the %EL_1slice_, 0.997 for the %EL_3slices_, and 0.997 for the %EL_volume_ in the cochlea, and 0.991 for the %EL_1slice_, 0.997 for the %EL_3slices_, and 0.996 for the %EL_volume_ in the vestibule.

In both observers, it took 15–20 minutes to contour the cochlea and the vestibule on all slices of the MRC for the right and left sides.

## Discussion

The degree of EHs on MR images has been graded using a previously proposed subjective scoring system in many studies.^11,16–18^ However, subjective grading using only three categories (i.e., significant, mild, and none) might not be sensitive enough to monitor subtle treatment effects.^10,11,14,19^ Quantification of EHs has been the goal of several studies.^3,11,20^ A highly sophisticated method has been proposed using high contrast data obtained by IT-GBCM.^13^ Even for the lower contrast images obtained by IV-SD-GBCM, a volume quantification method has been reported,^14^ however it required some observer-dependent and time-consuming ROI settings. In the present study, we showed the feasibility of estimating the %EL_volume_ from the %EL_3slices_ or the %EL_1slice_ with multiple observers.

The correlation coefficients of the %EL_3slices_ were slightly higher than that of the %EL_1slice_ in the cochlea and the vestibule for both observers. It is quite reasonable that a correlation coefficient increase would be observed by increasing the number of slices used in the measurement. The small difference in the correlation coefficients between the %EL_3slices_ and the %EL_1slice_, might allow for the simple estimation of the %EL_volume_ from the %EL_1slice_. A less time-consuming and simpler image evaluation method might increase the clinical feasibility of this method.

There are some limitations to this study. We did not include patients with inner ear anomalies. If the shape of the cochlea or the vestibule is deformed, a volume estimation from 1 slice or 3 slices might not be feasible. This retrospective single center study with a small number of patients might have included some patient selection bias. Although we controlled the ROI placement by carefully outlining a detailed instruction for contouring, the method used in the present study is still susceptible to observers’ ROI placement variability. An automatic bias free segmentation method should be developed in the future.^11^

## Conclusion

The endolymphatic volume ratio might be easily estimated from area ratio measurements on only 1 or 3 slices. This simple method might further promote the widespread use of quantitative MR imaging for the evaluation of EHs.

## Figures and Tables

**Table 1.** Pulse sequence parameters

Sequence name	Type	Repetition time (ms)	Echo time (ms)	Inversion time (ms)	Flip angle (degree)	Section thickness/gap (mm)	Pixel size (mm)	Number of slices	Echo train length	Field of view (mm)	Matrix size	Number of excitations	Scan time (min)
MR cisternography (MRC)	SPACE with restore pulse	4400	544	NA	90/ initial 180 decrease to constant 120	1/0	0.5 × 0.5	104	173	165 × 196	324 × 384	1.8	3
Heavily T_2_ weighted 3D-FLAIR (PPI)	SPACE with inversion pulse	9000	544	2250	90/ initial 180 decrease to constant 120	1/0	0.5 × 0.5	104	173	165 × 196	324 × 384	4	15

GRAPPA × 2 for all sequences. All sequences utilize a frequency selective fat suppression pre-pulse. Each three-dimensional slab is set in an identical axial orientation. PPI, positive perilymph image; SPACE, sampling perfection with application-optimized contrasts using different flip angle evolutions.
